# Nuclear functions of prefoldin

**DOI:** 10.1098/rsob.140085

**Published:** 2014-07-09

**Authors:** Gonzalo Millán-Zambrano, Sebastián Chávez

**Affiliations:** 1 Instituto de Biomedicina de Sevilla, Hospital Virgen del Rocío-CSIC-Universidad de Sevilla, 41013 Seville, Spain; 2 Departamento de Genética, Facultad de Biología, Universidad de Sevilla, 41012 Seville, Spain

**Keywords:** prefoldin, gene transcription, cytoskeleton, chromatin, protein folding

## Abstract

Prefoldin is a cochaperone, present in all eukaryotes, that cooperates with the chaperonin CCT. It is known mainly for its functional relevance in the cytoplasmic folding of actin and tubulin monomers during cytoskeleton assembly. However, both canonical and prefoldin-like subunits of this heterohexameric complex have also been found in the nucleus, and are functionally connected with nuclear processes in yeast and metazoa. Plant prefoldin has also been detected in the nucleus and physically associated with a gene regulator. In this review, we summarize the information available on the involvement of prefoldin in nuclear phenomena, place special emphasis on gene transcription, and discuss the possibility of a global coordination between gene regulation and cytoplasmic dynamics mediated by prefoldin.

## 2. Introduction

Misfolded proteins are detected shortly after their synthesis or after denaturation events, and are targeted to refolding. A large set of molecular chaperones and auxiliary factors are involved in this phenomenon (reviewed in [1]). Prefoldin was first described as a cochaperone [2], capable of capturing unfolded polypeptides and transferring them to the ATP-dependent class II chaperonin CCT [3], also known as c-cpn [4] or TriC [5].

**Figure 1.**
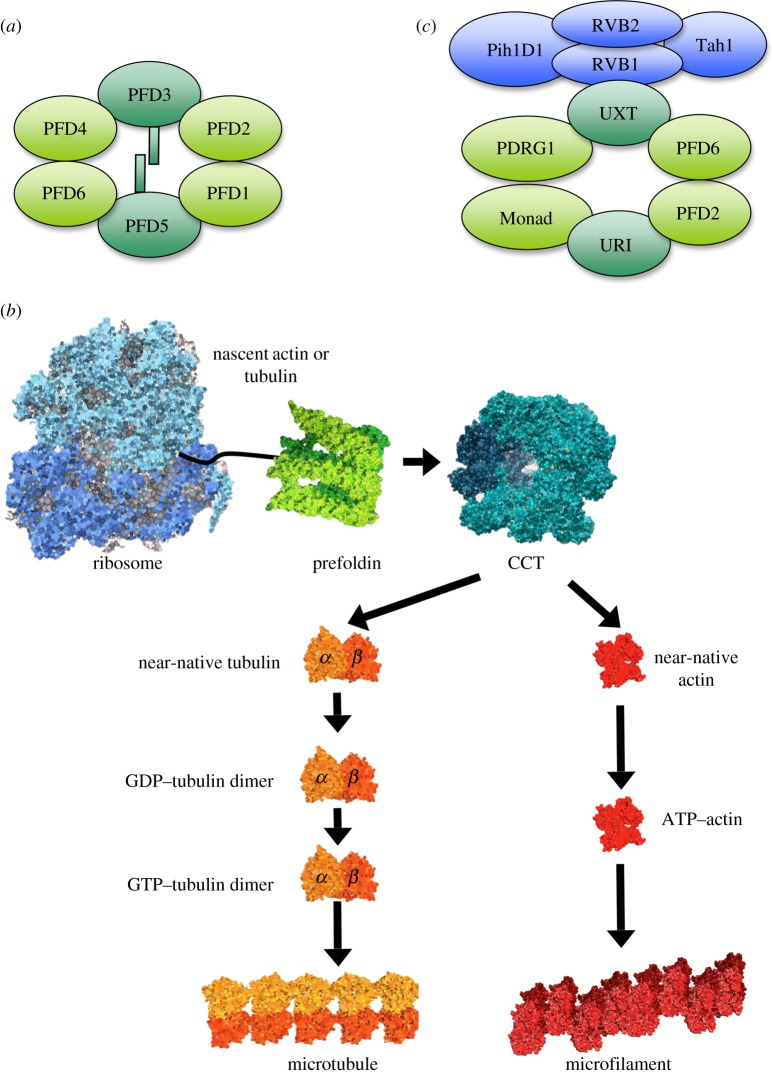
The prefoldin complex. (*a*) Canonical prefoldin is a heterohexameric complex composed of two α subunits (dark green), which play a central structural role, and four β subunits (light green). (*b*) The best-characterized function of prefoldin is the cotranslational folding of proteins. Prefoldin binds unfolded polypeptides and transfers them to the ATP-dependent chaperon CCT prior to its assembly into high-order protein structures, such as microtubules and actin filaments. (*c*) In addition to canonical complexes, which retain the structure of archaeal prefoldin, eukaryotes exhibit prefoldin-like complexes. In these, the two α and some of the β canonical subunits are replaced with alternative polypeptides. Prefoldin-like complexes interact and functionally cooperate with other cochaperones such as the R2TP complex (purple).

Prefoldin is not present in Eubacteria, but it is present in Archaea [6], suggesting that its ubiquitous presence in the eukaryotic kingdom is archaeal in origin [7]. Prefoldin is a heterohexameric complex. Whereas archaeal prefoldin is composed of two identical α and four identical β subunits, canonical eukaryotic prefoldin is composed of two different α and four different β subunits (figure 1*a* and table 1). All eukaryotic organisms, from yeast to human, share this heterohexameric structure, indicating an early differentiation of the prefoldin subunits during the evolution of eukaryotes. Eukaryotic prefoldin is evolutionarily conserved, because human and plant subunits functionally complement yeast prefoldin mutants [8,9]. In all cases, two subunits of the α class form a dimer, onto which four subunits of the beta class assemble and produce a jellyfish-like complex [10]. This complex consists of a double β barrel assembly with six long tentacle-like coiled coils protruding from it. The distal regions of these coiled coils expose hydrophobic patches, required for the binding of misfolded proteins. This feature situates prefoldin among the set of chaperone factors that use clamp-like structural features to grip substrate proteins [11].

**Table 1.** The canonical and prefoldin-like subunit nomenclature. Most prefoldin subunits have synonymic names. In order to facilitate comprehension, in this review, we have chosen the main nomenclature of metazoan prefoldin (PFDN1-6). Likewise, the mammalian prefoldin-like subunits have been named several times. We call them URI and UXT for the sake of simplicity.

Archaea	*S. cerevisiae*	higher eukaryotes
*β*	Pfd1/Gim6	PFDN1
	Pfd2/Gim4	PFDN2
	Pfd4/Gim3	PFDN4
	Pfd6/Gim1/Yke2	PFDN6/HKE2
α	Pfd3/Gim2/Pac10/Rks2	PFDN3/VBP1
	Pfd5/Gim5	PFDN5/MM1
	Bud27	URI/RMP
		UXT/Art-27

Cytoskeleton components are the best-known targets of eukaryotic prefoldin [8]. This captures unfolded actin, α- and β-tubulin cotranslationally, and remains bound to the relatively unfolded polypeptides until their posttranslational delivery to CCT [12] (figure 1*b*). Recognition of actin and tubulin by prefoldin involves specific interactions between certain domains of the target proteins [13] and the distal ends of different, but overlapping, sets of prefoldin subunits [14]. The so-formed prefoldin-target binary complex is then able to interact with CCT. In this ternary complex, prefoldin transfers actin to CCT following a handoff mechanism [15]. Interestingly, the interaction between actin and eukaryotic prefoldin maps inside the cavity formed by its six subunits, whereas archaeal prefoldin stabilizes unfolded proteins by interacting with the distal regions of the chaperone tentacles. This suggests that the substrate interaction mechanism of prefoldin has diverged through evolution and potentially reflects a narrower range of substrates stabilized by prefoldin in eukaryotes [16]. Consistent with a role played by prefoldin in actin and tubulin folding, the deletion of prefoldin-encoding genes in *Saccharomyces cerevisiae* results in impaired cytoskeleton functions [2]. These cytoskeletal defects of the prefoldin mutants are even more marked in the absence of the phosducin-like protein 3, a factor that physically interacts with CCT and modulates its chaperonin ATPase activity *in vitro* [17].

While none of the prefoldin subunits is essential for yeast viability, the mutation of prefoldin genes or the depletion of their products in *Caenorhabditis elegans* and *Drosophila* results in embryonic lethality. In both cases, prefoldin-defective cells show low tubulin levels and several cytoskeleton abnormalities [18,19]. In the nematode, prefoldin depletion leads to a lower microtubule growth rate [18] and prevents pronuclear migration [20]. *mgr* flies, which are mutated in the gene encoding PFDN3, exhibit circular mitotic figures and loss of meiotic spindle integrity [19]. In *Arabidopsis*, PFDN3, PFDN5 and PFDN6 are required for normal microtubule dynamics and organization [9,21], whereas the genetic murine models affected in PFDN1 and PFDN5 display developmental defects that were interpreted in terms of cilia and cytoskeleton defects [22,23].

All these pieces of evidence have shaped the eukaryotic prefoldin concept as a highly specialized cochaperone for actin and tubulin folding. This specialization would be consistent with the more severe phenotype of CCT knockdown than the prefoldin one in *C. elegans* [18]. However, a more in-depth review of the scientific literature reveals that eukaryotic prefoldin has also been connected to phenomena that are not directly linked to the cytoskeleton, such as protein aggregation. Prefoldin prevents protein aggregation in brain cells under conditions where protein degradation is compromised [24]. This role of prefoldin explains its protective effect against polyglutamine toxicity and the accumulation of aggregated pathogenic huntingtin [25]. Human prefoldin also inhibits amyloid-β fibrillation and contributes to the formation of non-toxic amyloid-β aggregates *in vitro*, which is consistent with its upregulation in a murine model for Alzheimer's disease [26] and the genetic association of prefoldin variants with this pathology [27].

The involvement of prefoldin subunits in the cytoplasmic assembly of some non-cytoskeletal complexes has also been well established. In this case, mammalian PFDN2 and PFDN6 form a complex together with UXT, RPB5, WDR92/Monad, PDRG1 and URI [28]. In this complex, which is supposed to adopt a prefoldin-like structure, non-canonical prefoldin proteins substitute for the α subunits and some of the β subunits (figure 1*c*), although no evident sequence similarity exists between canonical and non-canonical prefoldin polypeptides. The prefoldin-like complex participates in the cytoplasmic assembly of RNA polymerase II [29], and in the stabilization and assembly of phosphatidylinositol-3 kinase-related protein kinase [30]. In both cases, it cooperates with another cochaperone, the R2TP complex, which is composed of four subunits: RVB1, RVB2, PihD1 and hSpagh [31]. The latter two subunits of R2TP interact with the Hsp90 chaperone, whereas RVB1 and RVB2 are AAA+ ATPases that participate in a set of additional cellular activities. They act in the context of R2TP, such as in the maturation of small nucleolar ribonucleoprotein complexes, or independently, contributing to histone acetylation, chromatin remodelling, telomere dynamics and mitotic spindle assembly (for a review, see [32]). Functional analyses in mammalian cells indicate that the URI subunit of this complex is a target of nutrient signalling and participates in TOR kinase-controlled gene expression [28]. URI is conserved through the evolution of eukaryotes, and its yeast homologue Bud27 also mediates TOR-controlled gene expression [28] and is involved in the cytoplasmic assembly of all three yeast nuclear RNA polymerases [33].

α-class prefoldin-like subunit UXT is also located in human centrosomes, associates with γ-tubulin and its overexpression disrupts the centrosome structure [34]. This finding indicates that the canonical prefoldin complex may share some of its cytoskeleton functions with non-canonical prefoldin subunits.

## 3. Prefoldin shuttles between the cytoplasm and the nucleus, and acts on DNA-binding proteins

All the phenomena related to prefoldin that we have examined so far in this review take place in the cytoplasm. However, both the canonical and prefoldin-like subunits have also been found in the nucleus. Proteomic analyses of URI nuclear interactors indicate that all the components of the R2TP/prefoldin-like complex, including the canonical prefoldin subunits PFDN2 and PFDN6, are found in the nucleus of prostate cells, and shuttle between the nucleus and the cytoplasm, probably together with RNA pol II [35]. The short isoform UXT-V2/Art-27, of UXT, which has 157 amino acids, is primarily present in the nucleus, whereas UXT-V1, which encompasses 169 amino acids, is predominantly found in the cytoplasm [36,37]. This nuclear localization preference of some splice variants also happens in the case of human PFDN5/MM-1. MM-1β and MM-1δ localize mainly to the cytoplasm, whereas MM-1α and MM-1γ are found in the nucleus [38].

Canonical plant prefoldin, at least its subunits PFDN5 and PFDN6, is also found in the nucleus of *Nicotiana benthamiana* and *Arabidopsis thaliana* leaf cells [39]. Its nuclear localization is not constitutive, but dependent on the physical interaction with the nuclear DELLA proteins. Plant prefoldin moves from the nucleus to the cytosol in response to environmental or endogenous cues that cause degradation of DELLA proteins [39]. Human PFDN3, also known as von Hippel–Lindau binding protein1 (VBP1), because of its physical interaction with VHL, localizes to the cytoplasm when it is solely expressed, but it translocates to the nucleus when it is co-expressed with VHL [40]. In turn, VHL localization to the nucleus depends on UXT [41]. Localization of URI in the nucleus also depends of its interaction with a partner, in this case with the DNA methyltransferase 1-associating protein (DMAP1), which inhibits the cytoplasmic localization signal of URI and favours its import into the nucleus [42].

In addition to their cytoplasmic localization, all yeast canonical prefoldin subunits can be detected in the nucleus. Moreover, they accumulate in the nucleus when the nuclear export systems based on Xpo1 and Mex67 become genetically inactivated [43]. So, from yeast to metazoa, prefoldin subunits are capable of migrating to the nucleus and of being translocated back to the cytoplasm by active export systems.

**Figure 2.**
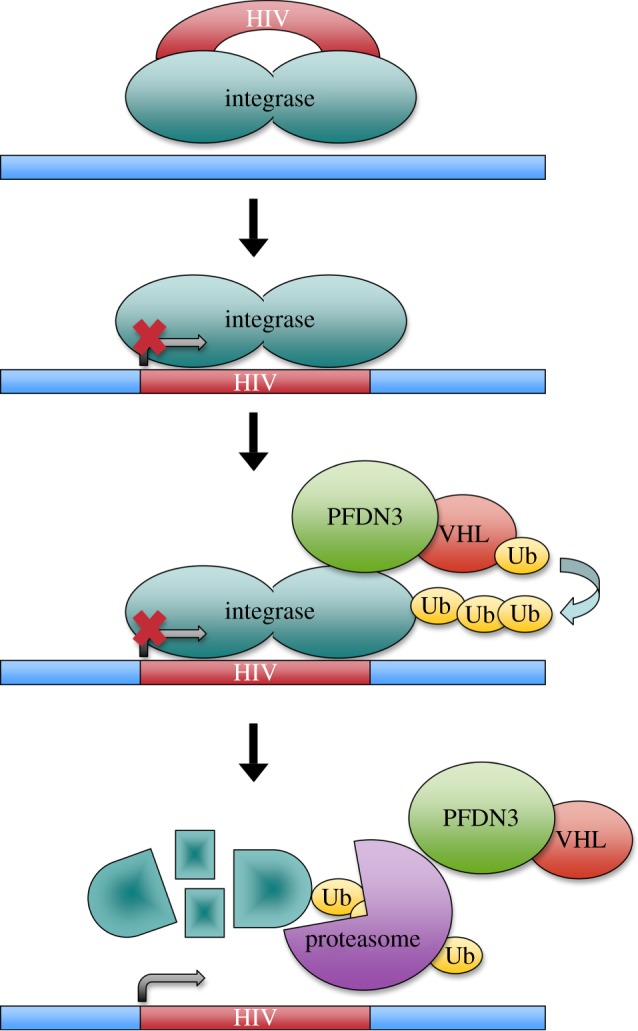
Human PFDN3 favours HIV transcription by driving its integrase into degradation. HIV integrase remains bound to proviral DNA after its integration into the host genome. PFDN3 is required for the *in situ* ubiquitination of the HIV integrase and its subsequent degradation by the proteasome, by virtue of its physical interaction with the E3-ubiquitin ligase VHL.

Localization of prefoldin in the nucleus may be the result of the regulation of its cytoplasmic functions by means of a cytoplasm-exclusion mechanism, as has been demonstrated in *Arabidopsis* [39]. In addition to this passive presence of prefoldin in the nucleus, several pieces of evidence indicate that the nucleus is not just a reservoir for stand-by prefoldin. URI, for instance, is required for DNA stability in *C. elegans* [44]. A clearer example of a nuclear function of prefoldin is its participation in the degradation of the HIV integrase after the provirus integration into the host genome, which is a strict requirement for HIV expression [45]. PFDN3 binds the integrase [46] and mediates its interaction with the cullin2-based VHL ubiquitin ligase, which is essential for its polyubiquitination and subsequent proteosome-mediated degradation [45] (figure 2). At least two other prefoldin subunits (PFDN1 and PFDN6) influence HIV expression at a post-integration step, suggesting that the whole prefoldin complex acts on the HIV integrase [45]. PFDN3 also facilitates the VHL-mediated degradation of other well-known DNA-interacting proteins, such as the DNA mismatch repair protein hMSH4 [47]. In this case, however, the co-localization of these two proteins is mainly perinuclear [48].

This nuclear role of mammalian PFDN3 in protein degradation is quite the opposite to that described for *Drosophila* PFDN3 in the microtubule dynamics context. In this case, PFDN3 promotes microtubule stabilization when tubulins are correctly folded by prefoldin, and tubulin destruction when they are not [19]. This double role of prefoldin in protein folding and degradation resembles that of protein segregases such as the VCP/p97/Cdc48 AAA-ATPase, which segregates ubiquitinated proteins from stable assemblies with proteins, membranes and chromatin [49]. Targets of VCP can either end in degradation, or can survive as free subunits and recycle [50]. No physical or functional connection between prefoldin and VCP has been established so far.

## 4. Prefoldin plays transcriptional roles

Among the nuclear proteins bound and influenced by prefoldin, transcription factors are the most frequent (table 2). Prefoldin-like subunit UXT binds the EVI1 transcriptional repressor and suppresses its cell transformation activity [59]. UXT is also an integral component of the NF-κB enhanceosome and is essential for its nuclear function [60]. Its knockdown leads to impaired NF-κB activity and dramatically attenuates the expression of NF-κB-dependent genes [60]. UXT is therefore an optimal target for NF-κB regulation. The Epstein–Barr virus BGLF4 kinase downregulates NF-κB transactivation by means of UXT phosphorylation, and the subsequent interference of this modification with the interaction between UXT and NF-κB [61]. Amyotrophic lateral sclerosis 2 protein has also been suggested to modulate the NF-κB pathway through its physical interaction with UXT [62].

**Table 2.** Nuclear proteins that physically interact with canonical or prefoldin-like subunits. For detailed explanations, see the text.

prefoldin subunit	interactor (biological process)	other factors involved	organism	reference
PFDN3	HIV integrase	VHL	human	[45]
	hMSH4 (DNA repair)	VHL, p97	human	[47]
	NF-κB (transcription)	HBx	human	[51]
PFDN5	c-Myc (transcription)	HDAC1–mSin3, TIF1*β*	human	[52,53]
		Skp2–ElonginB–ElonginC–Cullin2, Rabring7	human	[54,55]
	EGR1(transcription)		human	[56]
	p73 (transcription)		human	[57]
PFDN3, PFDN5	DELLA (gene regulation)		*Arabidopsis thaliana*	[39]
URI	HBx (transcription)	Rpb5	human	[58]
	DMAP1 (transcription)		human	[42]
UXT	EVI1 (transcription)		human	[59]
	NF-κB (transcription)		human	[60]
	HBV EGLF4 kinase (gene regulation)		human	[61]
	ALS2 (gene regulation)		human	[62]
	androgen receptor (transcription)	VHL	human	[36,41,63]
		URI	human	[35]
	LRP16 (transcription)		human	[64]
	TAF130 (transcription)		human	[36]
	Sp1 (transcription)		human	[36]

UXT is not only involved in NF-κB-dependent transcription regulation, but it also contributes to the regulation of androgen-dependent genes by binding the N-terminal domain of the androgen receptor (AR) and enhancing its transcriptional activation [36,63]. UXT also interacts with LRP16, a macrodomain-containing protein that functions as a coactivator of AR and other nuclear receptors [64]. The positive contribution of UXT- to AR-dependent transactivation seems to be mediated, at least partially, by the capacity of UXT to interact with VHL and to facilitate VHL-dependent ubiquitination of AR [41]. UXT interacts directly with VHL [41] and without the involvement of PFDN3, indicating that this ubiquitinating protein can interact with both canonical and prefoldin-like subunits. Two-hybrid assays suggest that UXT is also able to interact with the human transcription factor Sp1 and the TBP-interacting protein TAF130 [36].

A different prefoldin-like subunit, URI, can repress AR-mediated transcription by interacting physically with UXT in the chromatin context [35]. URI seems to inhibit AR recruitment to target genes because it is bound to chromatin prior to the hormonal activation of AR [35]. URI also inhibits transactivation by other gene-specific transcription factors, such as herpes simplex virus transactivator VP16 and hepatitis B virus protein X (HBx) [58]. In this case, URI inhibits HBx-activated transcription by competing with the activator to bind the RNA polymerase II subunit Rpb5 [58]. The co-repressor activity of URI is also related to its interaction with DMAP1, a partner of the histone deacetylase HDAC2 [42]. Interestingly, canonical prefoldin subunit PFDN3 also influences HBx transcriptional function, yet, in this case, it cooperates positively with this viral protein in the activation of the NF-κB transcription factor [51]. All these data indicate considerable plasticity in the ability of the different canonical and prefoldin-like subunits to interact with transcription factors, and suggest that they do not necessarily shape a single functional entity in the nucleus of mammalian cells.

**Figure 3.**
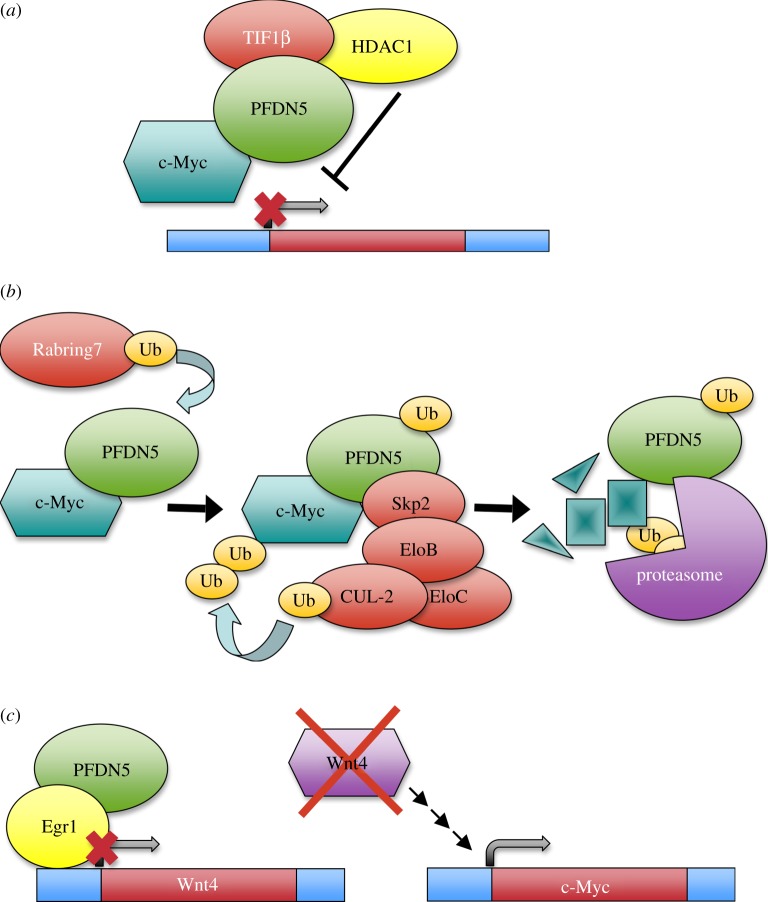
Human PFDN5 is involved in three different control mechanisms of c-Myc. (*a*) PFDN5 binds the N-terminal region of c-Myc, and represses its transcriptional activity by recruiting the TIF1β correpressor and the histone deacetylase HDAC1–mSin3 complex. (*b*) PFDN5 drives c-Myc into proteasome-dependent degradation by recruiting the ubiquitin ligase Skp2–ElonginC–ElonginB–Cullin2 complex. PFDN5 mono-ubiquitination, which is induced by Rabring7, stimulates this process. (*c*) PFDN5 and Egr1 cooperate in the transcriptional repression of Wnt4, which is one of the elements of the Wnt-β-catenin pathway that positively controls the c-Myc gene.

In addition to the degradation of HIV integrase, the best-characterized contribution of a prefoldin subunit to transcription is the action of PFDN5/MM-1 as a co-repressor of the E-box-dependent transactivation activity of c-Myc [52]. The MM-1α and MM-1γ nuclear isoforms bind the N-proximal region of c-Myc, which accommodates one of its transactivation domains, and repress its transcriptional activity [38]. A point mutation in PFDN5, which is often observed in patients with leukaemia or lymphoma, abrogates all of its repressive activities towards c-Myc, indicating that PFDN5 behaves like a tumour suppressor [65]. The repressive function of PFDN5 is due to its ability to recruit the histone deacetylase HDAC1–mSin3 complex via transcriptional co-repressor TIF1β. This recruitment antagonizes the histone transacetylases bound to the N-terminal domain of c-Myc, thereby inhibiting chromatin remodelling [53] (figure 3*a*). This inhibitory effect of PFDN5 on c-Myc offers a chance to modulate this transcription factor; a good example of it is the ability of the hepatitis C virus ARFP/F protein to enhance the gene transactivation activity of c-Myc by interfering in its interaction with PFDN5 [66].

The negative action of PFDN5 on the c-Myc function also takes place at two other levels. It favours c-Myc degradation by recruiting the ubiquitin ligase Skp2–ElonginC–ElonginB–Cullin2 complex, and driving it to the proteasome via the 26S subunit Rpt3 [54] (figure 3*b*). The monoubiquitination of PFDN5 by Rabring7, a Rab7-binding and RING finger-containing protein, stimulates this second role of PFDN5 in the control of c-Myc [55] (figure 3*b*). In addition, PFDN5 and the Egr-1 repressor bind and downregulate the promoter of the wnt4 gene. Because the c-Myc gene is the target of the Wnt–β-catenin pathway, PFDN5 also inhibits the expression of c-Myc by this indirect mechanism [56] (figure 3*c*).

**Figure 4.**
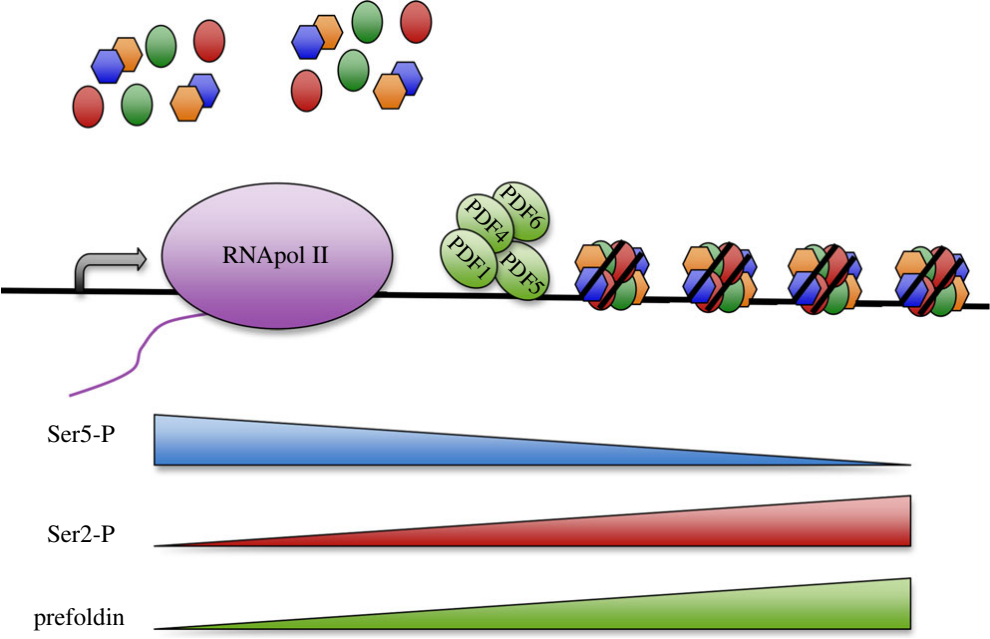
Yeast prefoldin stimulates transcription elongation by favouring chromatin dynamics. A subset of canonical prefoldin subunits, including PFDN1, 4, 5 and 6, binds transcribed genes in a transcription-dependent manner and contributes to transcription elongation. This effect is maximal in long genes, where prefoldin stimulates cotranscriptional histone eviction. Prefoldin binding is absent in the promoter region and is maximal in the 3′ end of the gene body to correlate with the presence of Ser2-hyperphosphorylated RNA polymerase II.

The above-described interactions indicate a role of prefoldin subunits in the regulation of the transactivation capacity or stability of gene-specific transcription factors. With the exception of its contribution to HIV expression, the transcriptional roles of prefoldin have been described for single subunits. Although the presence of other prefoldin subunits cannot be ruled out, the mechanisms described so far do not involve the action of prefoldin complexes. *Saccharomyces* prefoldin, however, seems to play a transcriptional role that involves at least the concerted action of PFDN1, PFDN2, PFDN5 and PFDN6 [43] (figure 4). These four canonical prefoldin subunits are recruited together to yeast transcribed genes in a transcription-dependent manner. The profile of recruited prefoldin parallels the phosphorylation of the Ser2 residues of Rpb1 CTD, a well-known marker of RNA polymerase II elongation activity. Accordingly, the deletion of any of the genes encoding these four prefoldin subunits provokes transcription elongation defects [43]. In agreement with this role of yeast prefoldin in transcription elongation, genes longer than 4 kpb are particularly affected. The absence of prefoldin increases the density of histones that remain bound to these genes under intensive transcription. However, nucleosome remodelling, reflected in the sensitivity of chromatin to micrococcal nuclease digestion, remains unaffected by the absence of prefoldin. Together, these pieces of evidence indicate a role of prefoldin in histone eviction after the cotranscriptional destabilization of nucleosomes [43]. This prefoldin–chromatin connection is fully consistent with the strong genetic interactions detected between prefoldin and a wide set of chromatin factors [67,68]. Interestingly, the yeast CCT mutants display the very same pattern of genetic interactions with chromatin factors [69]. Moreover, mouse CCT exhibits a nucleocytoplasmic distribution and associates to heterochromatin [70], and depletion of Cct2 interferes with HIV integration at a post-integration step, exactly as prefoldin does [45]. Taken altogether, the available experimental evidence suggests that prefoldin and CCT might also cooperate in the transcriptional dynamics of chromatin.

## 5. Final perspective: prefoldin in global cell regulation

To date, we have shown that the scientific literature contains a significant amount of information on the presence of canonical and prefoldin-like subunits in the nucleus, and their functional involvement in gene transcription. The examples that we cite demonstrate that, from yeast to mammals, a given prefoldin subunit can play cytoplasmic and nuclear roles in the same organism. Moreover, a set of yeast canonical subunits plays concerted actions in both cytoplasmic cytoskeleton assembly and nuclear gene transcription. This fact might merely be the result of a functional divergence during the evolutionary transition from archaea to eukaryotes. Alternatively, the cytoplasmic and nuclear functions of prefoldin might be coupled in such a way as to favour a global coordination of gene expression and cytoskeleton dynamics. This hypothetical coordination is particularly expected in those situations where cells are challenged by stimuli that require a specific genomic response and, at the same time, the reorganization of cytoplasmic organelles. Although this speculative model is far from having been demonstrated, there are some experimental hints that are compatible with the notion of prefoldin as a global cell regulator.

The cytoskeletal function of prefoldin is not essential for the housekeeping assembly of microtubules or actin filaments. This is the reason why yeast prefoldin genes can be deleted without compromising cell viability [8]. Similarly, double knockout mice, lacking PFDN1, are also viable, at least until the fifth week of age, although they display a small size and cytoskeletal defects [22]. When β-tubulin is not expressed at high levels in *Drosophila* cells, its stabilization does not require prefoldin either, although it does when ectopic β-tubulin is expressed [19]. This indicates that prefoldin is rate-limiting only under strong cytoskeleton biogenesis conditions. Rate-limiting steps are the regulatory points in most biological pathways, and they are usually the most upstream stages. Accordingly, prefoldin plays its role in the very first step of microtubules and actin filaments biogenesis [18].

One of the most drastic cytoplasmic reorganizations takes place during B-lymphocyte activation by antigens, when they differentiate into highly secretory plasma cells. PFDN1 knockout mice are severely affected in this process. Interestingly, the mammalian PFDN6 gene is located in the centromeric portion of the class II region of the major histocompatibility complex [71], and its expression dramatically increases as a result of lymphocyte activation [72].

**Figure 5.**
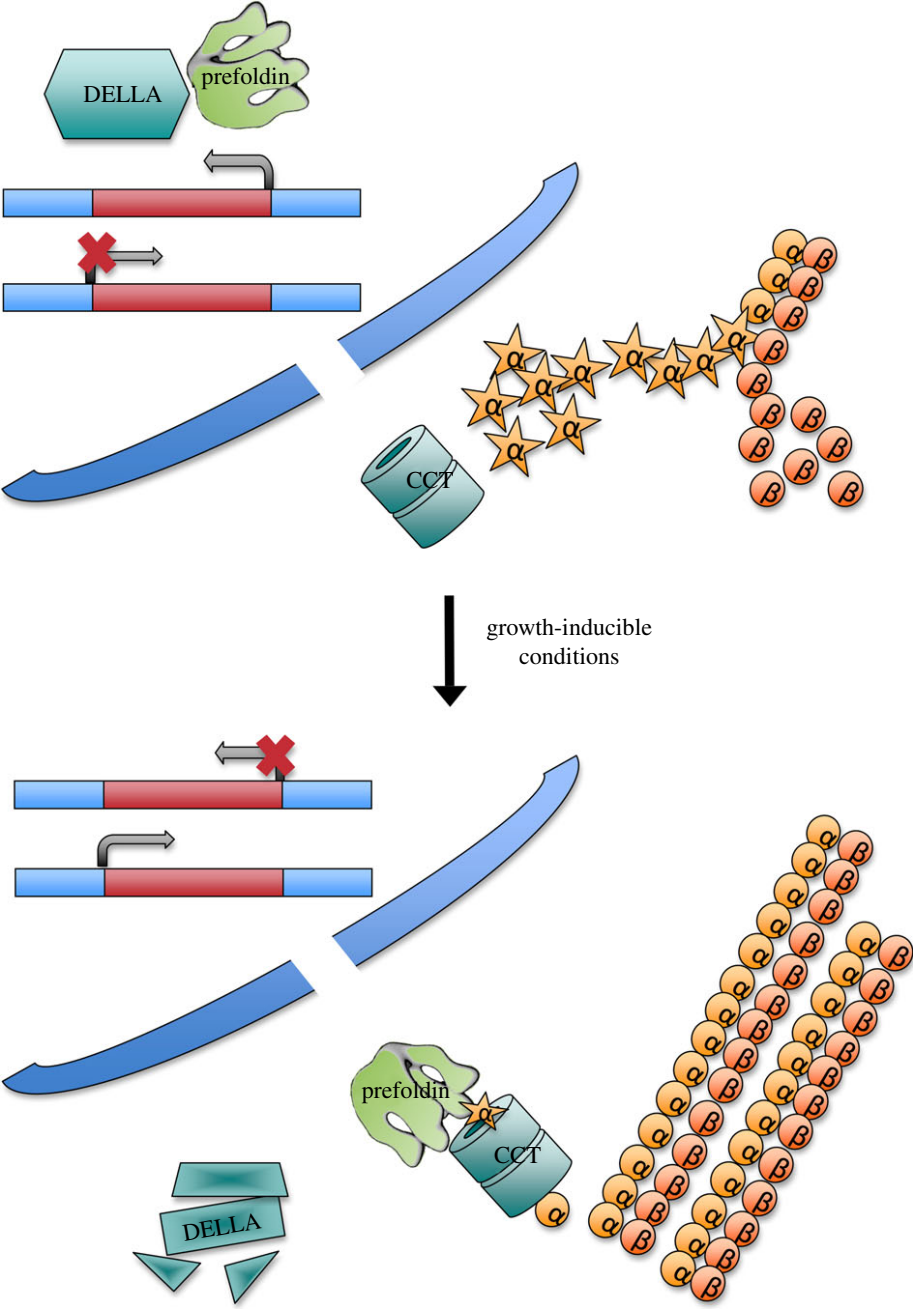
Plant prefoldin collaborates with the DELLA transcription factor in the regulation of cell expansion. When environmental conditions are not favourable for growth, prefoldin is imported to the nucleus by the DELLA transcription factor, which contributes to the regulation of a set of genes, either positively or negatively. Upon activation by growth-inducible conditions, DELLA is driven into degradation, allowing prefoldin to relocate to the cytoplasm and to participate in the cytoskeleton reorganization required for cell expansion.

So far, the prefoldin role in lymphocyte activation has not been connected to any nuclear event, but there is one example where the cytoplasmic function of prefoldin is linked to the nucleus. This is the case of organ growth by cell expansion in the model plant *Arabidopsis thaliana*. The plant controls anisotropic cell elongation by a mechanism that finely coordinates the abundance of DELLA proteins in the nucleus with the subcellular localization of prefoldin [39]. When environmental conditions are not favourable for growth, DELLA proteins accumulate, and prefoldin is retained in the nucleus upon interaction, compromising its role in the cytoplasm. Thus, microtubule assembly is compromised, and anisotropic cell growth is prevented. On the contrary, environmental conditions that are favourable for growth promote degradation of DELLA proteins and prefoldin can move to the cytosol (figure 5) [39]. DELLAs are transcriptional regulators and, although no transcriptional function of prefoldin has been described to date in *Arabidopsis*, it is possible that prefoldin participates not only in the cytoplasmic part of this regulated process, but also in the transcriptional response.

The coordinated action in cytoskeleton dynamics and transcription would be facilitated if prefoldin acted on similar targets in these two processes. It is well known that the main cytoplasmic targets of prefoldin are actin and tubulin monomers. It is conceivable that actin, tubulin or both might also be nuclear targets of prefoldin. The transcriptional characterization of yeast prefoldin mutants, using chemical inhibitors of cytoskeleton assembly, indicates that the role of prefoldin in chromatin dynamics during transcription elongation is not mediated by actin or tubulin polymerization [43]. Yet the involvement of actin and tubulin monomers in the nuclear function of prefoldin is an open possibility. There is monomeric actin in the nucleus, and its contribution to gene transcription has already been reported (reviewed by [73]). The direct involvement of nuclear tubulin in gene transcription has also been described [74]. c-Myc, the best example of a transcription factor regulated by a prefoldin subunit (see above), also binds tubulin, and the region of the c-Myc protein that interacts with tubulin overlaps its PFDN5 interaction domain [75]. It has been proposed that microtubules might be involved in the migration of c-Myc to the cytoplasm, when cells exit the cell cycle [76,77]. Myc-nick, a cleavage product of c-Myc, promotes tubulin acetylation during cell differentiation [78], and MIZ-1, another c-Myc-interacting protein, is regulated by its association to microtubules and activate transcription in response to cytoskeleton changes [79]. All these data visualize physical and functional interactions between c-Myc and tubulin, which support functional coupling between c-Myc-dependent transcription and the cytoskeleton dynamics mediated by prefoldin.

The existence of such mechanisms of coordination between cytoplasmic dynamics and genome regulation, and the potential involvement of prefoldin and other protein-folding factors in them, is a challenging field for contemporary biology.
